# After 25 years of computer-navigated total knee arthroplasty, where do we stand today?

**DOI:** 10.1186/s42836-021-00100-9

**Published:** 2021-11-04

**Authors:** Siddharth M. Shah

**Affiliations:** S. L. Raheja (A Fortis Associate) Hospital, Raheja Rugnalaya Marg, Mahim West, Mumbai, Maharashtra 400 016 India

**Keywords:** Computer navigation, Computer-assisted, Robotic, Total knee arthroplasty, Total knee replacement, Alignment, Outliers

## Abstract

**Background:**

Limb and implant alignment along with soft tissue balance plays a vital role in the outcomes after total knee arthroplasty (TKA). Computer navigation for TKA was first introduced in 1997 with the aim of implanting the prosthetic components with accuracy and precision. This review discusses the technique, current status, and scientific evidence pertaining to computer-navigated TKA.

**Body:**

The adoption of navigated TKA has slowly but steadily increased across the globe since its inception 25 years ago. It has been more rapid in some countries like Australia than others, like the UK. Contemporary, large console-based navigation systems help control almost every aspect of TKA, including the depth and orientation of femoral and tibial resections, soft-tissue release, and customization of femoral and tibial implant positions in order to obtain desired alignment and balance. Navigated TKA results in better limb and implant alignment and reduces outliers as compared to conventional TKA. However, controversy still exists over whether improved alignment provides superior function and longevity. Surgeons may also be hesitant to adopt this technology due to the associated learning curve, slightly increased surgical time, fear of pin site complications, and the initial set-up cost. Furthermore, the recent advent of robotic-assisted TKA which provides benefits like precision in bone resections and avoiding soft-tissue damage due to uncontrolled sawing, in addition to those of computer navigation, might be responsible for the latter technology taking a backseat.

**Conclusion:**

This review summarizes the current state of computer-navigated TKA. The superiority of computer navigation to conventional TKA in improving accuracy is well established. Robotic-assisted TKA provides enhanced functionality as compared to computer navigation but is significantly more expensive. Whether robotic-assisted TKA offers any substantive advantages over navigation is yet to be conclusively proven. Irrespective of the form, the use of computer-assisted TKA is on the rise worldwide and is here to stay.

## Introduction

Alignment of components in total knee arthroplasty (TKA) influences function and implant longevity [1–3]. Although, the ideal alignment principle in TKA is debatable, every surgeon wishes to implant the components in a desired orientation with accuracy and precision. The resultant need has given birth to computer-assisted navigation in TKA. The first computer-navigated TKA was reportedly performed in 1997 [4]. The early system consisted of a computer, optical localizer, and arrays mounted with light emitting diodes (LEDs) which could be attached to bones and surgical instruments to aid in the desired placement of cutting blocks [4]. Although, the basics remain the same, the technology has undergone extensive validation and upgrades over the years to provide the enhanced functionality of current generation of computer navigation systems.

Aseptic loosening (31.2%) and instability (18.7%) are the two commonest modes of failure of TKA [5]. Alignment and soft tissue balance during surgery can influence both of these factors [1, 6, 7]. The number of TKAs performed globally is rising. Kurtz et al [8] estimated a 673% increase in primary TKA from 2005 to 2030 in the United States. Total knee arthroplasties are also being increasingly performed in the younger population. These patients are likely to place higher demands on their prosthesis; revision rates amongst the younger population are projected to be higher [9]. Hence, achieving optimum alignment and soft-tissue balance becomes all the more important, especially in the younger patients. Computer navigation might help address this need.

### Adoption of computer navigated TKA

The use of computer navigation for TKA has slowly but steadily increased since its inception. The spread of this technology has not been uniform across the globe. Its adoption has been more rapid in some countries compared to others. In Australia, the proportion of computer-navigated TKA rose from 2.4% in 2003 to 32% in 2019 [10]. In a survey of European Society of Sports Traumatology Knee Surgery and Arthroscopy (ESSKA) and Swiss Orthopaedic Society members, Friederich and Verdonk [11] reported that 51.9% members had a navigation system at their centre and 25.7% surgeons used it for more than 75% of their TKAs. In Germany, more than 30% surgeons use computer technology for TKA [12]. In contrast, Antonios et al [13] reported that the proportion of computer-navigated TKA increased from 1.2% in 2005 to only 6.3% in 2014, in the United States. The proportion of computer-navigated TKA in the UK is estimated to be less than 3% of all TKAs [12].

According to the ‘Diffusion of innovations’ theory by Everett Rogers, adopters of new technology belong to these categories: innovators, early adopters, early majority, late majority, and laggards; and the process of adoption over time is demonstrated by a bell curve of normal distribution [14]. Computer-navigated TKA is in the ‘early adopter’ phase of Roger’s bell curve of technology adoption cycle. Despite clear demonstration that it significantly improves component alignment compared to conventional TKA, computer-navigated TKA has not become mainstream yet. Picard et al [12] looked into the reasons for the same which are discussed herewith. Some of the earlier systems were perceived to be ergonomically unsound and lacked user-friendliness which was an important reason for resistance to adopting this technology [12]. However, most computer navigation systems today are intuitive and easy to use. Another factor is the economic aspects related to it. There is an initial cost of acquiring the system. There may be recurring costs of single use reflective spheres used on the arrays. Depending upon the healthcare model, the associated costs may be passed on to the patient/their insurance provider or absorbed by the state. The increased cost associated with navigated TKA can be offset by savings accrued due to reduction in revision rates. Novak et al [15] looked into the cost-effectiveness of computer-assisted TKA and reported that cost savings can be achieved if the additional cost due to computer navigation was ≤629 $ per operated case. Their findings were based on a decision-analysis model using model inputs obtained from a review of literature. However, this analysis is also influenced by the variability in the cost of the system, the accuracy of the alignment obtained using navigation, and the probability of future revision surgery due to malalignment, as stated by the authors [15]. In reality, the analysis of cost-effectiveness can be very complex as it does not account for factors like increased surgical time and its secondary effects which are difficult to quantify.

Increased surgical time due to the use of computer navigation may also discourage surgeons from switching over from conventional TKA. Computer-navigated TKA may require between 10 and 20 extra minutes as compared to conventional TKAs. Bolognesi et al [16] reported an additional mean tourniquet time of 11 min with computer-navigated TKA as compared to conventional TKA. Surgeons may also be concerned about the learning curve associated with the computer-navigated TKA. The reported learning curve for navigated TKA is between 16 and 20 cases [17, 18]. Also, ‘disruptive innovations’ like minimally invasive surgery and patient-specific instrumentation, which did not clearly demonstrate any significant benefits, but distracted surgeons away from ‘sustaining’ technologies like computer navigation were responsible, to some extent, for navigated TKA not becoming mainstream [12].

Abundant scientific literature is available about navigated TKA. While the evidence unequivocally shows that it results in significant improvement in limb and component alignment as compared to conventional TKA [19–21], conflicting data exist regarding functional improvement and longevity which could be a reason why many surgeons would refrain from adopting this technology [19, 22–29]. Total knee arthroplasty using robotic arm or robotic hand-held devices has been introduced in the last 5 years. While navigation systems help accurate placement of cutting blocks, which is followed by freehand cutting of bones, robotic systems help precise sculpting of bones to place the implants in a desired orientation. Although, robotic systems are significantly more expensive than navigation systems, they provide enhanced functionality and may reduce alignment errors due to inaccurate bone cuts. Most leading orthopedic companies have already launched or are in the process of launching robotic TKA platforms. Understandably, in such a scenario, the industry is more likely to focus more on the propagation of this newer, robotics-based technology rather than computer navigation.

### Classification

**Fig. 1:**
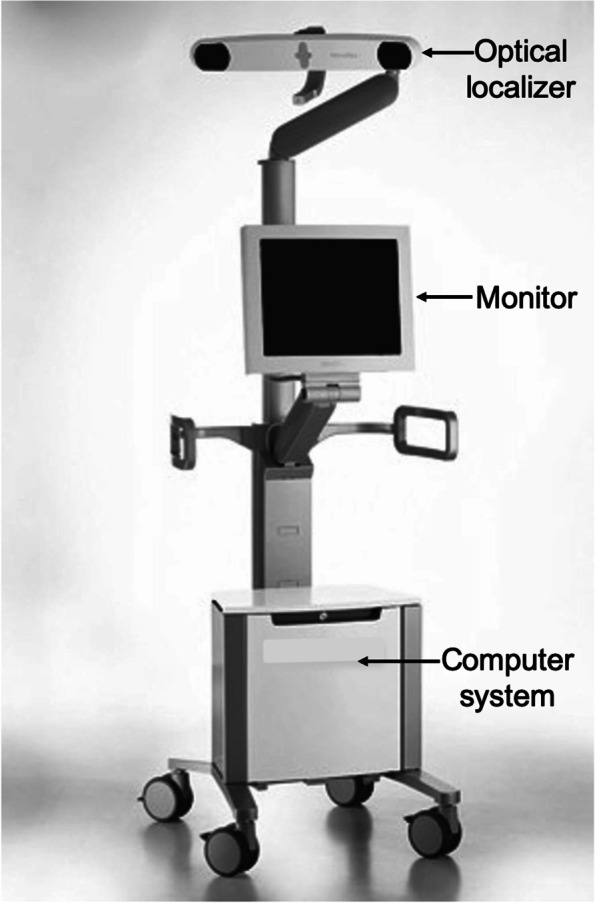
A contemporary computer navigation system displaying its main parts: optical localizer, monitor, and computer system

Jones and Jerabek [30] classified computer navigation systems into large-console navigation systems (Fig. 1) and accelerometer-based hand-held navigation systems. Large-console navigation systems could be image-based or imageless. Image-based systems require a preoperative CT scan to build a frame of reference. They are not popular due to the additional cost and radiation exposure to the patient. Imageless navigation systems use intraoperative data to build the reference frame and are the most commonly used systems today.

### Basics of large-console navigation systems

**Fig. 2:**
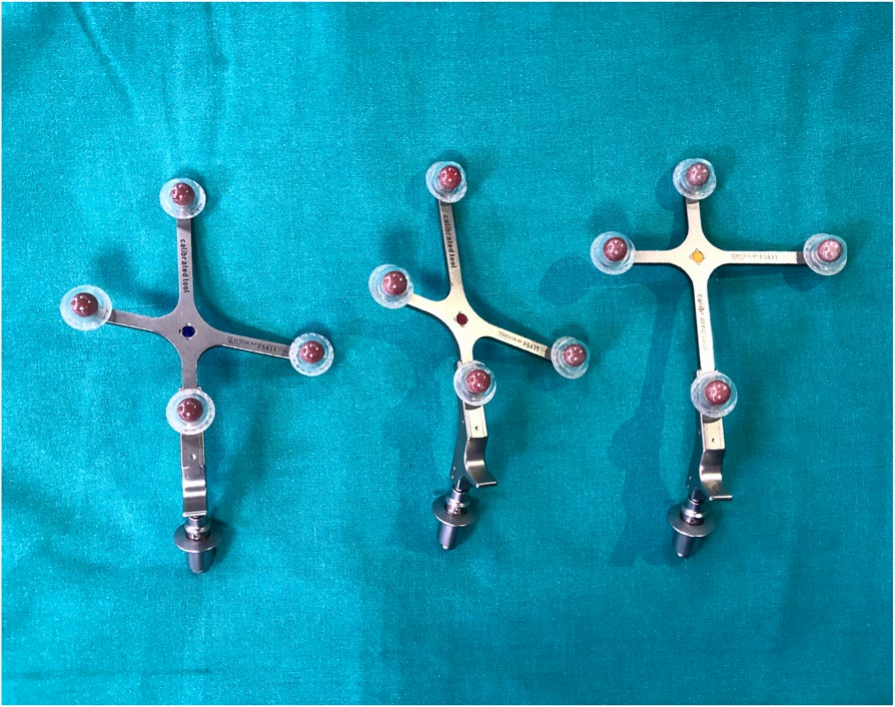
Passive trackers with reflective spheres

The essential components of a computer navigation system are trackers/arrays (Fig. 2), localizer (Fig. 1), and computer system. During surgery, trackers are attached to the bones, surgical instruments, or a probe. They can be ‘passive’ in the form of reflective spheres (Fig. 2) or ‘active’ with LEDs. The localizer or camera receives reflected (passive tracker) or active (active tracker) signals from the trackers to determine their spatial orientation. The most commonly used localizers are ‘optical’ localizers. The downside of optical localizers is that the trackers must always be within the localizer/optical field to be detected. In contrast, electromagnetic (EM) systems have a receiver which can detect signals from the trackers without requiring them to be in the line of sight. Lastly, the ‘brain’ is the computer system which processes information received by the localizer to provide real-time 3-D graphical data about alignment and knee joint morphology to the surgeon throughout surgery.

### Steps of computer-navigated TKA

Most modern navigation systems allow the surgeon to customize the surgical workflow according to their preferences. Options include the choice of ‘measured resection’ or ‘gap-balancing’. With ‘measured resection’, the surgeon may choose the ‘tibia’ or ‘femur first’ technique. Additional options include the choice of anatomical landmarks for setting femoral rotation. Some systems may also offer optional steps to determine medio-lateral coverage of femoral component and setting of tibial rotation.

**Fig. 3:**
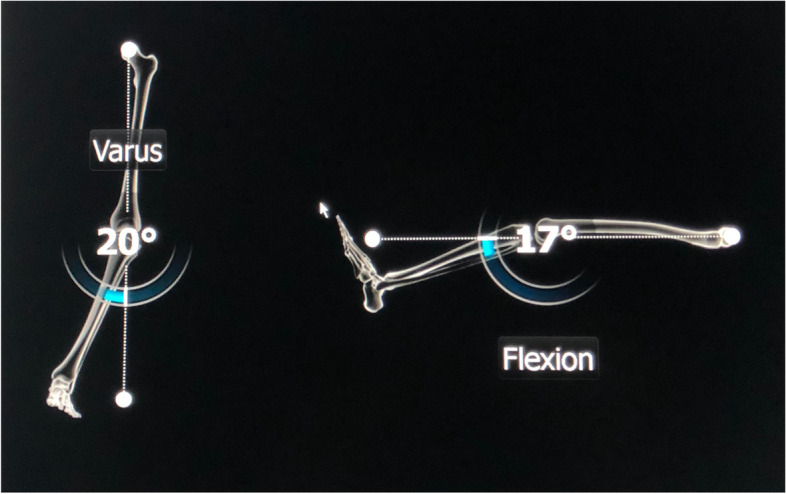
Computer monitor displaying initial limb alignment in coronal and sagittal planes

**Fig. 4:**
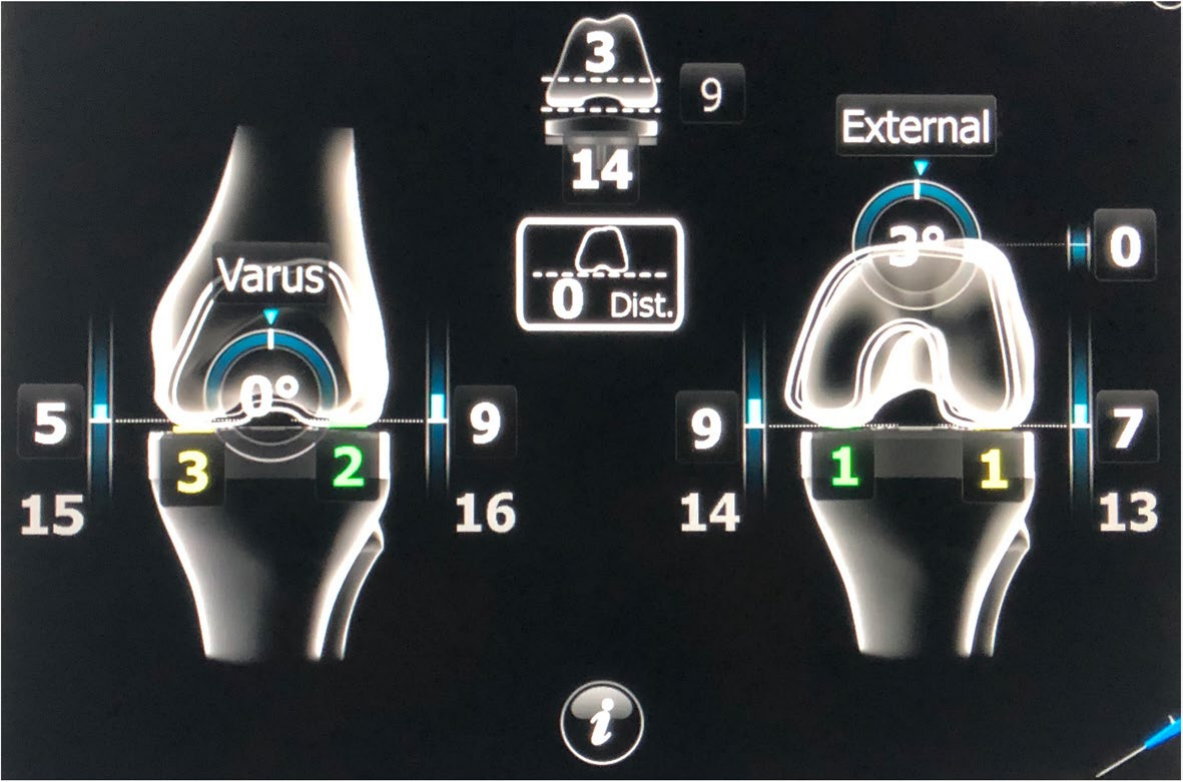
Screenshot displaying customization of femoral component in order to obtain desired gap balance in extension (left) and 90° flexion (right). One can customize femoral size, depth of distal femoral resection (joint line), orientation of distal femoral cut in coronal (varus/valgus) and sagittal (flexion/extension) planes, femoral rotation, and insert size

The navigation console is commonly placed contralateral to the side of the knee to be operated upon. After a standard exposure of the knee joint, the trackers are attached to the lower end of the femur and upper end of tibia using two-pin uni-cortical or a single-screw bi-cortical fixation. The trackers must be so attached that they are within the localizer field throughout the surgery and do not interfere with placement of surgical instruments. This is followed by the registration process which involves acquisition of centers of the hip, knee, and ankle joints and surface mapping of the distal femur and proximal tibia. Registration is a critical step as the computer system uses this data to build the frame of reference and inaccuracies during this process will result in incorrect data being displayed by the system, eventually leading to surgical errors. The popular computing phase ‘garbage in, garbage out’ (GIGO) is apt here. Modern navigation systems have built-in redundancies which can alert the surgeon if there is a significantly large error while acquiring certain landmarks during the registration process. Once the registration is completed, the system provides information about limb alignment (Fig. 3) and joint morphology which the surgeon can use to determine the orientation and depth of bone resections, titrate soft-tissue releases, and customize implant position to obtain desired alignment and soft tissue balance. One can control the orientation and depth of proximal tibial and distal femoral resections, adjust femoral size, and set femoral rotation (Fig. 4). Newer versions of some navigation systems now also allow setting of tibial component rotation. Thus, the system allows 3-D customization of implant position based on surgeon’s preference. After the respective bone resections, one can also verify them and re-cut to the desired level, if required. Most contemporary systems also provide data about joint kinematics and balance throughout the range of motion before and after bone cuts. If ‘gap-balancing’ protocol is chosen, medial and lateral knee joint gaps in extension and 90° flexion are displayed which the surgeon can use to plan soft tissue releases and adjust the femoral implant position to achieve desired alignment and balance.

### Computer navigation and pin site complications

Uni- or bi-cortical trans-osseous pins are used to attach the trackers to the lower end of femur and upper end of tibia during computer-navigated TKA. The possibility of pin tract complications is often discussed and may discourage surgeons from adopting this technique. These could be in the form of pin tract infection, pin site pain, heterotopic ossification (rare), and fracture (rare). In a systematic review looking into pin site fractures after computer-navigated or robotic knee replacements, Smith *et al* [18] reported an incidence from 0.06 to 4.8% [31]. Risk factors for pin site fractures included transcortical pin placement, diaphyseal placement, pin diameter size > 4 mm, non-self-drilling/non-self-tapping pins, and multiple drill holes [31]. In my experience of several hundred navigated TKAs, I have not encountered a single fracture related to pin insertion.

### Accelerometer-based navigation (ABN)

Hand-held ABN is basically a system of sensors which are attached to cutting guides or bones in order to aid in the placement of cutting blocks in a desired position. They were launched with the aim of overcoming some of the drawbacks of large-console systems. Compared to large-console systems, its advantages include no initial set-up cost, no ‘line of sight’ problems associated with optical tracking, avoidance of pin site complications as it does not require trans-osseous femoral and tibial tracker fixation, and reduced surgical time [32]. However, ABN systems do not provide information about soft-tissue tension and cannot aid in setting femoral or tibial rotations. Also, there is an additional cost of single-use components at the time of surgery.

### Scientific literature

There is evidence that suggests navigated TKA results in significantly better implant and limb alignment as compared to conventional TKA. In a meta-analysis of 23 randomized controlled trials, Hetaimish et al [21] reported that navigated TKA had significantly lower risk of implant malalignment at both ±3° and ± 2° limits. In a meta-analysis of 47 studies, Moskal et al [20] demonstrated that navigated TKA significantly improved component alignment. In a meta-analysis of six studies comparing navigated and conventional TKA in patients undergoing bilateral surgery, Zhao et al [19] reported that navigated TKA resulted in better correction of lower limb mechanical axis and better prosthesis alignment. Mooney et al [33] showed that navigation-enhanced instrumentation significantly reduced the total outlier rate (±2°/2 mm) as compared to conventional instrumentation. Outliers (±3°) in terms of implant and limb alignment do occur after navigated TKA with a reported incidence of 10.4% [34] and can be attributed to the surgeon or the navigation system. Errors or inaccuracies during the registration process, especially during the registration of femoral epicondyles, will provide erroneous data to the surgeon which may result in malalignment [35].

In conventional TKA, unlike navigated TKA, an intramedullary rod is introduced into the femoral canal for determining the distal femoral cut. This breach of the femoral medullary canal may increase the risk of complications. Navigated TKA results in significantly lower blood loss as compared to conventional TKA [20, 36] and has significantly lesser need for perioperative transfusions [37]. Kalairajah et al [38] found significant reduction in systemic emboli with navigated TKA as compared to conventional TKA. Siu et al [39] reported significantly lower D-dimer levels and only milder surge in D-dimer levels, 24 h after navigated TKA as compared to conventional TKA. In contrast, Kim et al [40] found no significant differences in prevalence of embolic phenomenon between navigated and conventional TKA. Kuo et al [41] showed that navigated TKA results in lesser vascular injury as compared to conventional TKA. It also results in lower local and systemic inflammation [42]. These benefits are attributable to the fact that the femoral medullary canal isn’t breached during navigated TKA.

While certain advantages of navigated TKA are evident, controversy exists regarding other benefits. With the ability to obtain a desired implant alignment and better soft-tissue balance, one would expect that navigated TKA would provide better clinical and functional outcomes which has been reported by many authors [22, 25, 26, 29]. However, others found no significant difference between navigated and conventional TKA in terms of functional outcomes [19, 23, 24]. Implant and limb alignment can influence implant longevity. While individual studies and meta-analyses may have shown that navigated TKA did not result in improved implant survival [19, 23, 24], registry data were contrary to that. Data from the Australian Joint Replacement Registry showed that navigated TKA significantly reduced the overall revision rate and revision rate for loosening/lysis in patients < 65 years [27]. Jorgenssen et al [43]reported a significantly higher rate of major aseptic revision in non-navigated TKAs (hazards ratio: 1.32, *P* < 0.001) based on data from the Australian Joint Replacement Registry. Dyrthovden et al [28] reported similar survival and revision rates between navigated and conventional groups, but significantly reduced revision for malalignment in the navigated group, based on Norwegian registry data.

Literature regarding outcomes of ABN systems is available but not as extensive as large-console based navigation systems. In a meta-analysis of eight studies, Li et al [44] showed that one ABN system (iAssist) significantly improved lower limb alignment as compared to conventional TKA. However, the surgical time was prolonged with no significant difference in short-term functional outcomes. In a randomized controlled trial, Nam et al [45] showed that the KneeAlign ABN system decreased outliers in tibial component alignment as compared to conventional TKA using an extramedullary guide. A version of the same system was also found to be accurate for distal femoral resection during TKA [46]. The KneeAlign-2 system was found to be as accurate as large-console based navigation systems but resulted in significantly shorter tourniquet time [32].

## Discussion

Since its introduction in 1997, computer-navigated TKA has come a long way. It is clearly evident that navigation can help achieve desired implant and limb alignment accurately and in a predictable manner. Whether improved alignment results in better function and longevity is a matter of contention. Every surgeon, however, would like to align and balance the knee during TKA in an optimal manner for which computer navigation, indisputably has a role.

Certain situations where the utility of navigation cannot be over-emphasized are TKA with extra-articular deformities of the femur or tibia, retained hardware in the distal femur, and knees with ipsilateral long stem hip replacements. During conventional TKA, the distal femoral cut is taken with the aid of an intramedullary (IM) rod. The above clinical situations preclude placement of an IM rod. Some systems provide the option of a short or a ‘variable length’ IM rod to mitigate this situation. However, shorter length of the rod may affect the accuracy of the distal femoral cut. Computer navigation is extremely useful in these situations as the system determines the femoral mechanical axis using the hip and knee joint centers, and does not require insertion of an IM rod. The surgeon can then place the distal femoral cutting block in the desired orientation in relation to the femoral mechanical axis. Computer navigation also obviates the need for hardware removal and avoids excessive tissue dissection, thus offering a less invasive solution in such situations.

Despite accurate placement of cutting guides, errors may occur due to inaccurate bone cuts [47, 48]. Damage to important structures around the knee like the ligaments and tendons may occur due to improper or uncontrolled sawing of bones. Robotic-assisted TKA can help overcome these problems by ensuring accurate bone cuts to the desired resection levels without damaging the surrounding soft tissue structures. Both computer navigation and robotics belong to the same class of ‘computer-assisted’ surgical technology. While navigation systems are passive, robotic systems may be haptic-based, semi-active (constrained system under surgeon’s control) or active (capable of performing a task without surgeon’s intervention). As is the case with other surgical specialties, robots are making inroads into the clinical practice of arthroplasty. The additional prospect of accurately controlling bone cuts along with the benefits of computer navigation is undeniably attractive to a surgeon. This, however, comes at an increased expense as robotic systems cost many more times that of large-console navigation systems. In the region where the author is based, the cost of robotic systems ranges between 800,000 to 1,300,000 US dollars (USD) as compared to 75,000 to 130,000 USD for large-console navigation systems. Whether this additional cost translates into clinical, functional, and survival superiority of robotically implanted knee implants remains to be seen. It is no secret that orthopedic companies are presently focusing on the relatively new market of robotics and computer navigation may have taken a backseat.

## Conclusion

This review summarizes the current state of computer-navigated TKA. The superiority of computer navigation over conventional TKA in improving accuracy is well established. Robotic-assisted TKA provides enhanced functionality as compared to computer navigation but is significantly more expensive. Whether robotic-assisted TKA offers any substantive advantages over navigation is yet to be conclusively proven. Irrespective of the form, the use of computer-assisted TKA is rising worldwide and it is here to stay.

## Data Availability

Not applicable.

## Abbreviations

TKA: Total knee arthroplasty
LED: Light emitting diode
EM: Electromagnetic
ABN: Accelerometer based navigation
IM: Intramedullary
USD: US Dollar
